# Does Clostridium Perfringens Epsilon Toxin Mimic an Auto-Antigen Involved in Multiple Sclerosis?

**DOI:** 10.3390/toxins17010027

**Published:** 2025-01-07

**Authors:** Marie-Lise Gougeon, Valérie Seffer, Cezarela Hoxha, Elisabeth Maillart, Michel R. Popoff

**Affiliations:** 1 Unité Immunité Innée et Virus, Institut Pasteur, 25-28 rue du Dr. Roux, 75724 Paris, Cedex 15, France; valerie.seffer@pasteur.fr; 2 Unité des Toxines Bactériennes, Institut Pasteur, Université Paris Cité, CNRS UMR 2001 INSERM U1306, 75015 Paris, France; cezarela.hoxha@pasteur.fr (C.H.); michel-robert.popoff@pasteur.fr (M.R.P.); 3 Département de Neurologie, AP-HP, Hôpital Pitié-Salpêtrière, Multiple Sclerosis Center, 75015 Paris, France; elisabeth.maillart@aphp.fr

**Keywords:** multiple sclerosis, *Clostridium perfringens*, epsilon toxin ETX, IgM, IgG, IgA antibodies, EDSS

## Abstract

Multiple sclerosis (MS) is a chronic immune-mediated neurological disorder, characterized by progressive demyelination and neuronal cell loss in the central nervous system. Many possible causes of MS have been proposed, including genetic factors, environmental triggers, and infectious agents. Recently, *Clostridium perfringens* epsilon toxin (ETX) has been incriminated in MS, based initially on the isolation of the bacteria from a MS patient, combined with an immunoreactivity to ETX. To investigate a putative causative role of ETX in MS, we analyzed the pattern of antibodies reacting to the toxin using a sensitive qualitative assay. This prospective observational study included one hundred patients with relapsing remitting multiple sclerosis (RRMS), all untreated, and ninety matched healthy controls. By assessing the isotypic pattern and serum concentration of ETX-reacting antibodies, our study shows a predominant IgM response over IgG and IgA antibody responses both in MS patients and controls, and significantly higher levels of IgM reacting to ETX in MS patients compared to the control group. A longitudinal follow-up of ETX-specific antibody response in a subgroup of MS patients did not show any correlation with disease evolution. Overall, these unexpected findings are not compatible with a specific recognition of ETX by serum antibodies from MS patients. They rather argue for a cross immunological reactivity with an antigen, possibly an autoantigen, mimicking ETX. Thus, our data argue against the hypothesis of a causal relationship between *C. perfringens* ETX and MS.

## 1. Introduction

Multiple sclerosis (MS) is considered as a chronic immune-mediated disease of the central nervous system (CNS). MS commonly affects adults between the ages of 20 and 50 years, and more often women (female to male sex ratio about 3:1) [1,2]. MS is characterized by progressive inflammation, demyelination, and neuronal cell loss in CNS [1,3,4]. Pathogenesis typically involves perivenular inflammation, blood brain barrier (BBB) opening, demyelination, and a late phase of tissue repair lasting for weeks to months [4,5]. T cells, notably CD4^+^ T cells, are considered as the main players in the genesis of MS lesions. The immunological processing in MS is further supported by increased levels of cytokines in the serum and cerebrospinal fluid (CSF) of patients. A balance between pro-inflammatory and anti-inflammatory cytokines seems crucial for the progression of the disease [6,7,8,9]. Several factors can trigger and/or modulate the immune system and subsequent inflammatory responses. Thus, changes in gut microbiota have been observed in MS patients. Gut dysbiosis can alter the intestinal permeability and facilitate the passage of microbial antigens, leading to immunomodulation and neuroinflammation in CNS [10,11,12,13]. However, the precise pathways from gut to neuroinflammation are still poorly understood and the initial cause of MS remains mysterious. A comprehensive description of the aetio-pathogenesis remains to be presented, but environmental factors such as smoking, vitamin D, and certain viral infections such as Epstein–Barr virus (EBV), now firmly established as a risk factor for MS [14], and rubella virus, also associated with increased risk of MS [15], are involved in disease development. Multiple candidate antigens include myelin autoantigens, notably the myelin basic protein and proteolipid protein, neuron- and astrocyte-derived antigens, viral antigens, and various gut microbial antigens or metabolites [8,13,14].

More recently, *Clostridium perfringens* epsilon toxin (ETX) has been incriminated in MS. An ETX-producing *C. perfringens* type B strain has been isolated from a MS patient, and immunoreactivity to ETX was found 10 times more prevalent in people with MS than in healthy controls (HCs) [16]. Another study showed that anti-ETX antibodies were about twice as frequent in MS patients than in the control group [17]. ETX-producing *C. perfringens* was detected more frequently in the gut microbiota of MS patients than in HCs, but at very low levels [18]. ETX is a pore-forming toxin produced by *C. perfringens* type B and D [19,20,21]. ETX is responsible for an acute and rapidly fatal disease in sheep, goats, and more rarely cattle, termed enterotoxemia [19,22,23]. Overgrowth of ETX-producing *C. perfringens*, mainly *C. perfringens* type D, in the intestine of these animal species results in secretion of high levels of ETX, which crosses the intestinal barrier without enterocyte alteration, disseminates through blood circulation, passes across the BBB, and targets specific cells in CNS such as granule cells, oligodendrocytes, and brain endothelial vascular cells [24,25,26]. This leads to excessive release of glutamate associated to neurological symptoms of convulsions and opisthotonos, and to brain lesions of perivascular edema and necrosis, which are observed in sheep enterotoxemia [27]. In experimental animals, mouse and rat, sublethal doses of ETX induce a demyelination [18,26,28] and ETX-induced experimental autoimmune encephalomyelitis has been proposed as a suitable model of MS [18]. ETX recognizes the myelin and lymphocyte (MAL) protein receptor, which is expressed in several tissues in humans, but most of them are not affected in MS [29,30]. Thus, a possible role of ETX in MS is questionable [31]. To further investigate if ETX is associated with MS, we analyzed anti-ETX antibodies in the sera of MS patients and healthy controls by qualitative and quantitative tests.

## 2. Results

### 2.1. Study Population

Demographics and clinical features are summarized in Table 1. The MS cohort included 100 patients with relapsing remitting MS (RRMS), all untreated at time of sampling. The median age was 36 years, IQR = 30–42, and 77% of MS patients were females. The mean EDSS score was 4.0 ± 1.4 (range 0–7.5), and the mean duration of disease was 9.3 ± 6.6 years (range 1 month–32.5 years). The dynamics of the different parameters and their possible relationship with the expansion or reduction of ETX-specific antibody response were studied in a 3- to 7-year period in a group of 10 patients, whose clinical characteristics are summarized in Table 1. Samples from 90 HCs were used to characterize serum reactivity to ETX in healthy subjects. For univariate analysis of indicated variables between MS and HC, sex- and age-matched controls were used from this cohort.

### 2.2. Immunological Response Against ETX as Tested by Western Blotting

**Figure 1:**
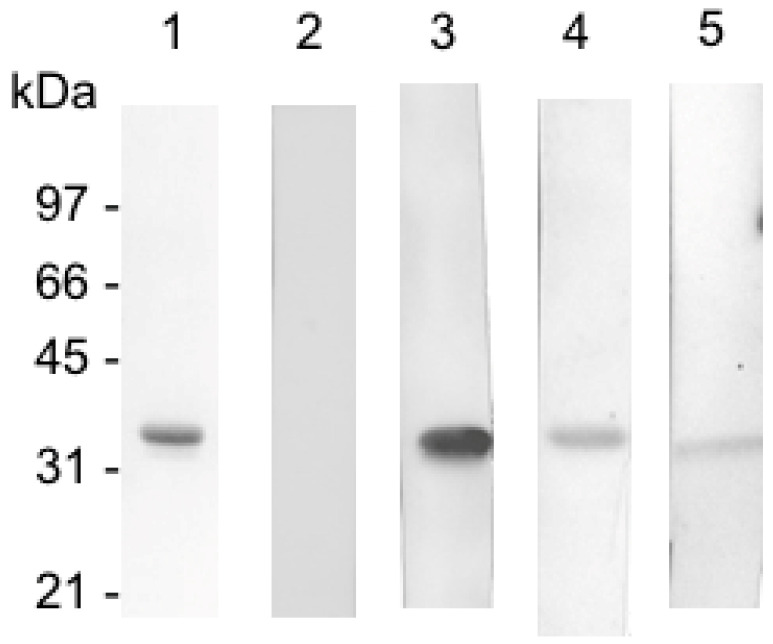
Western blots showing different immunoreactivities of sera to ETX. Lane 1, activated ETX (2.5 μg) stained with Coomassie blue. Lane 2, no reactivity with ETX. Lane 3, strong, Lane 4, medium, and Lane 5, weak immunoreactivity to ETX.

Sera from MS patients were tested by Western blotting with native ETX and 1000-fold serum dilution, as used by Wagley et al. [17]. Typical patterns of immunoreactivity of the sera are shown in Figure 1.

We found that 65.1% of 89 MS sera reacted with ETX as well as 77.7% of healthy controls (Table 2). The strong immune-reactive responses to ETX were slightly similar in both populations. Most MS patients showed a weak immuno-reactivity against ETX, whereas a medium response was found in the sera of healthy controls. However, the distinction between weak and medium reactivity in Western blotting is subjective and not highly precise.

### 2.3. Absence of Immunological Neutralization Response Against ETX

Any of the sera from MS patients (n = 100) and HCs (n = 90) showed a neutralization activity against ETX, as tested at 1:10 dilution in a MDCK cytotoxicity assay monitored by the entry of propidium iodide (PI) (cf. Section 4).

### 2.4. ETX-Specific Antibody Response in Healthy Controls and MS Patients

**Figure 2:**
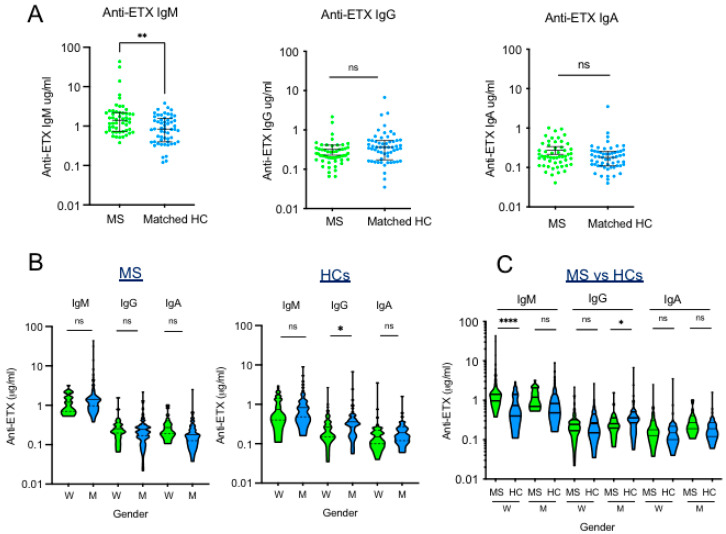
ETX-specific antibody response in MS patients vs. healthy controls. (**A**) Comparison of ETX-reacting IgM, IgG and IgA antibody responses in MS vs. matched HCs. (**B**) Comparison of ETX-reacting IgM, IgG, and IgA antibodies in MS and HCs according to gender. (**C**) Comparison of ETX-reacting IgM, IgG, and IgA antibody responses in MS vs. matched HCs according to gender. * *p* < 0.05; ** p < 0.01; **** p < 0.0001; ns, not significant.

To obtain a more precise picture of ETX immunoreactivity in human sera, we used a quantitative ELISA to determine the concentrations of ETX-specific antibodies, as well as their IgM, IgG, and IgA isotypes. We tested sera from 90 HCs and 100 MS patients. Figure 2A shows that the great majority of sera from HCs exhibited antibodies that reacted with ETX, and all three isotypes were detected. The breadth of ETX antibody response was high, varying from 0.1 to 10 μg/mL for IgM, and 0.05 to 5 μg/mL for IgG and IgA. The IgM antibody response was predominant and significantly higher than the IgG and IgA responses. A very similar pattern of antibodies reacting with ETX was detected in samples from MS patients. The median concentration of IgM reacting with ETX (1.42; IQR: 0.92–2.15 μg/mL) was significantly higher than the median concentration of IgG (0.25; IQR: 0.17–0.32) and IgA (0.19; IQR: 0.14–0.32), and it reached very high levels, ranging from 0.38 μg/mL to 43.0 μg/mL. Comparison of ETX-reacting antibodies in MS samples vs. sex- and age-matched healthy controls shows that only the IgM levels were significantly higher in MS patients compared to HCs (MS: median 1.41 μg/mL; IQR: 0.73–2.20 vs. HCs: median 0.84 μg/mL; IQR: 0.40–1.57, *p* < 0.001) (Figure 2A).

Considering that women are more at risk for MS than men and that they account for 77% of our cohort, we addressed the question of the impact of gender on ETX immunoreactivity. Analysis of the data, stratified according to gender within each group, showed that in both MS patients and HCs, the magnitude of ETX-specific IgM and IgA responses was similar in women compared to men, while IgG response was significantly increased in men (Figure 2B). When ETX immunoreactivity was compared between MS patients and HCs after gender stratification, a strong and highly significant increase in ETX-specific IgM response was detected in women with MS as compared to control women, whereas no difference was detected in men (Figure 2C). IgG and IgA responses were similar in women with MS vs. control women.

### 2.5. ETX-Specific Antibody Response According to Age

**Figure 3:**
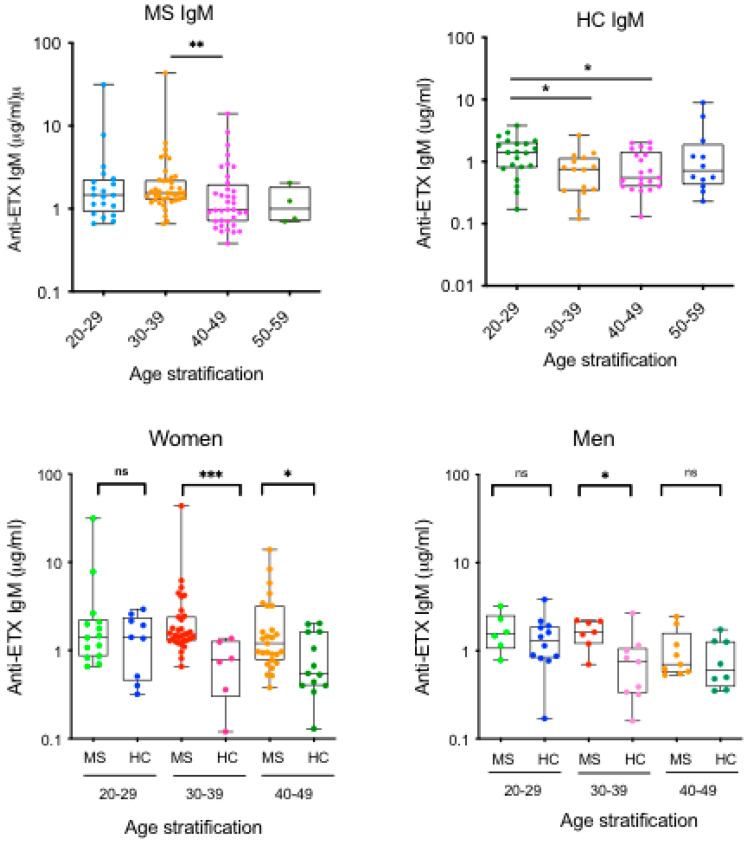
ETX-specific antibody response according to age. (**A**) Serum concentration of ETX-reacting IgM in MS patients and HCs according to age. (**B**) Comparison of serum concentration of ETX-reacting IgM in MS vs. HCs according to age in women and men. * *p* < 0.05; ** *p* < 0.01 *** *p* < 0.001; ns, not significant.

The onset of MS most commonly occurs between 20 and 40 years. It was therefore worthwhile analyzing a possible association between ETX-specific responses and age. Age stratification shows that IgM response in MS patients was at the highest level at the age of 30 to 39, then slowly declined while maintaining a consistent level (Figure 3A). This was not observed for ETX IgG or IgA responses. A different pattern was observed in HCs with a significantly higher IgM level at the age of 20 to 29 (Figure 3A).

We then focused our analyses on females and asked whether the levels of ETX-IgM would be increased in MS vs. HCs for specific age groups. Figure 3B shows that for both 30–39 and 40–49 age groups, IgM reacting with epsilon toxin were significantly higher in MS compared to HCs (*p* < 0.001 and *p* = 0.03, respectively). The same analysis performed on males shows a significant increase in IgM reacting with epsilon toxin for the 30–39 age group (*p* = 0.03) only, compared to HCs (Figure 3B). Altogether, these data highlight that MS is associated with significantly increased levels of IgM reacting with *C. perfringens* ETX, particularly detected in young women.

### 2.6. Reactivity of MS Sera with a Linear Peptide Spanning the Amino Acid Sequence of Epsilon Toxin

**Figure 4:**
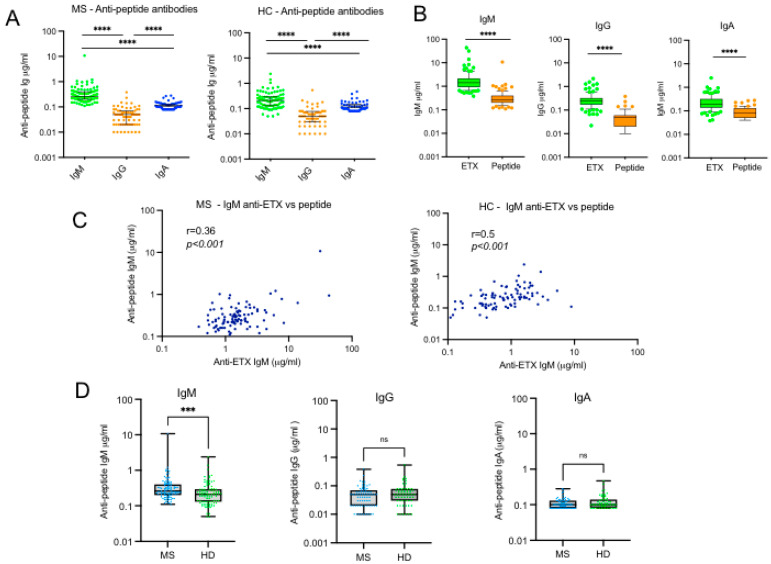
Reactivity of MS sera with ETX linear peptide TGVSLTTSYSFANTN. (**A**) Serum concentration of peptide-reacting IgM, IgG, and IgA antibodies in MS patients (left panel) and HCs (right panel). (**B**) Serum concentration of IgM, IgG, and IgA antibodies reacting to peptide vs. ETX in MS. (**C**) Linear regression of correlation between ETX vs. peptide IgM response in MS patients and HCs. (**D**) Comparison of peptide-reacting IgM, IgG, and IgA responses in MS vs. HCs. *** *p* < 0.001; **** *p* < 0.0001; ns, not significant.

In their study investigating for serum antibodies against ETX in UK patients with MS, Wagley et al. tested the sera for reactivity with linear overlapping peptides spanning the amino acid sequence of ETX. They found antibodies directed against the membrane insertion loop of domain 2 and especially against the TGVSLTTSYSFANTN peptide in sera from MS patients but not in sera from controls [17]. We tested the reactivity of sera from MS patients and HCs to this peptide. Figure 4A shows that the majority of sera, whether from patients or controls, reacted to this peptide, with a predominant IgM response, significantly higher than IgG and IgA responses. As expected, peptide-specific antibody response was significantly lower than ETX-specific response for the three isotypes (Figure 4B). However, a strong correlation was found between peptide-specific and ETX-specific antibody responses in sera from both MS patients and HCs, but this was observed only for IgM (Figure 4C). Such a correlation was not observed for IgG and IgA responses. Comparison of MS vs. HCs responses to the peptide shows that the level of IgM reacting with epsilon toxin peptide was significantly higher in MS compared to HCs (*p* < 0.001) (Figure 4D), as observed for ETX-specific antibody responses (Figure 2A). In contrast, peptide-specific IgG and IgA responses were similar in MS and HCs (Figure 4D).

### 2.7. Correlation Between ETX-Specific Antibody Response and MS Disease Evolution

**Figure 5:**
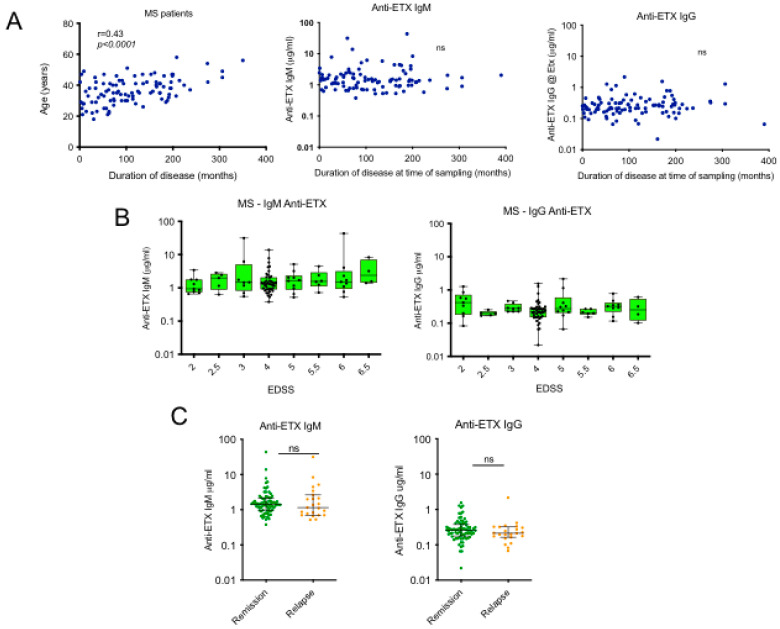
ETX-specific antibody responses and MS disease evolution. (**A**) Linear regression of correlation between age, ETX IgM, ETX IgG, and duration of disease evolution in all samples from MS patients. (**B**) Evolution of ETX-specific IgM (left panel) and IgG (right panel) responses according to EDSS stratification. (**C**) Comparison of anti-ETX IgM and IgG responses in MS patients in remission vs. relapse. ns, not significant.

To understand the meaning of increased levels of ETX-specific IgM in sera from MS patients, we took into consideration several parameters characterizing disease evolution, such as duration of disease, EDSS, and occurrence of relapses. While duration of disease was positively correlated with age (*p* < 0.0001), no correlation was found between disease evolution and ETX-specific IgM or IgG serum concentrations (Figure 5A). Similarly, no correlation was found between peptide-specific IgM and disease evolution. The expanded disability status scale (EDSS) is an ordinal clinical rating scale ranging from 0 (normal neurologic examination) to 10 (death due to MS). In the study MS patients, EDSS ranged from 0 to 7.5. Figure 5B shows that anti-ETX IgM or IgG levels did not vary with EDSS increase, arguing against a correlation between antibody response to epsilon toxin and EDSS. Among 100 MS patients diagnosed with RRMS, 33 had a relapse at time of sampling. Figure 5C shows that the levels of ETX-specific IgM or IgG did not differ between patients in remission and patients experiencing a relapse within this subgroup population.

### 2.8. Longitudinal Evolution of ETX-Specific Antibody Response in Relation with Disease Evolution

**Figure 6:**
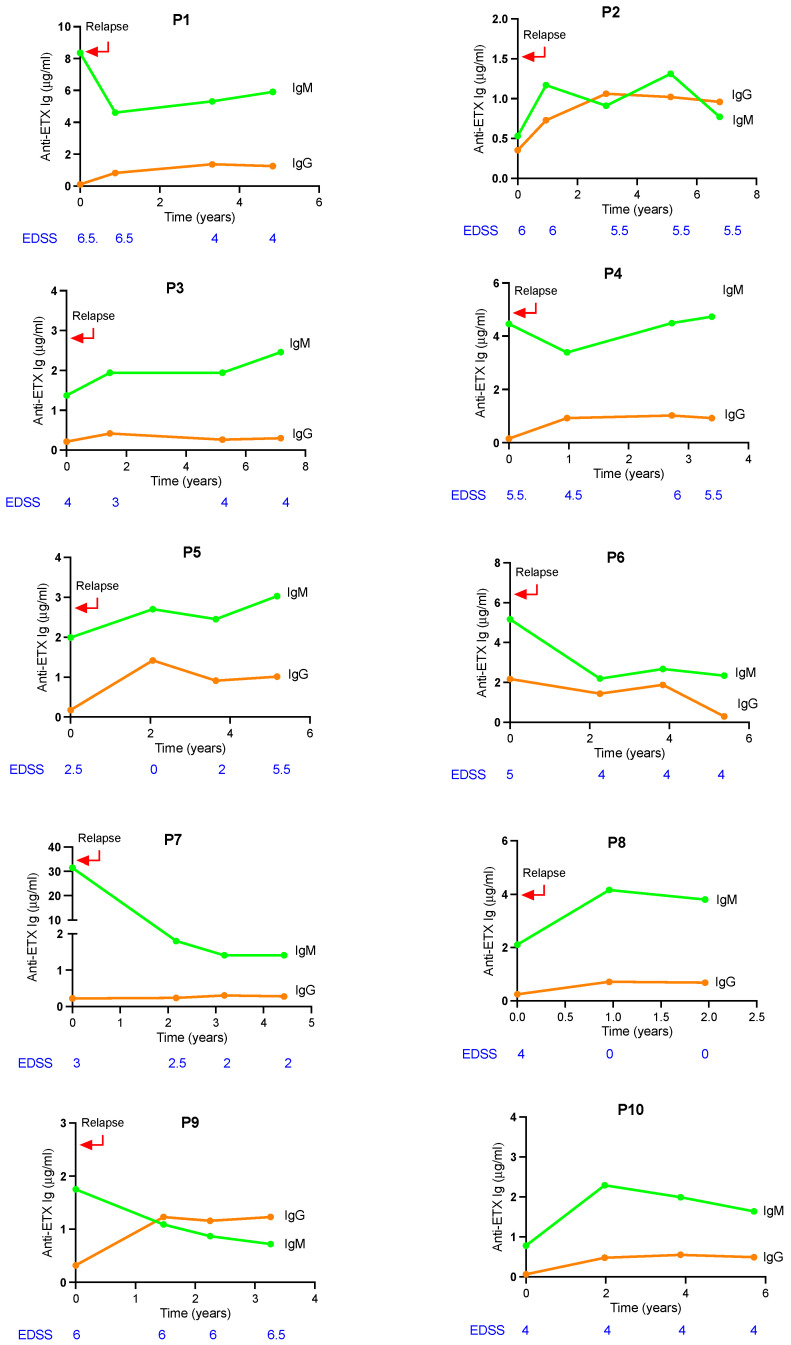
Longitudinal evolution of ETX-specific antibody response in relation to disease evolution. Prospective longitudinal study over a 2 to 5-year follow-up for 10 patients (P1 to P10). Baseline sampling was performed during a relapse, except for P10. The evolution of anti-ETX IgM and IgG responses, combined with EDSS, is shown over time from baseline to 2 to 5-year of follow-up.

A prospective longitudinal study could be performed over a 2 to 5-year follow-up for 10 patients. Longitudinal serum samples were available at baseline and at three follow-up visits. Clinical characteristics of these patients (P1 to P10) are detailed in Table 1. They were all female, non-treated RRMS, ranging from 21 to 47 years old, whose duration of disease varied from 6 months to 16.7 years, and EDSS ranged from 2.5 to 6.5 at sampling. Except for P10, baseline sampling was performed during a relapse. ETX-specific IgM and IgG antibody response kinetics, combined with EDSS, are shown in Figure 6. At baseline, the levels of IgM reacting with ETX were variable, ranging from 0.53 to 31.5 μg/mL, including levels exceeding 4 μg/mL for four patients (P1, P4, P6, P7), and even reaching > 30 μg/mL for one patient (P7). Apart from one exception (P2), IgG levels at baseline were much lower than IgM levels. Two profiles emerged regarding the kinetics of ETX-specific IgM. A rather flat response curve was observed for P2, P3, P4, P5, P8, and P10, while a steady decline was observed for P6, P7, and P9. We analyzed the relationship between changes in IgM levels and EDSS. In P6 and P7, the decline over time of IgM was associated with a slight decrease in EDSS. A transient decrease of IgM could be associated with a decrease in EDSS (P2, P4), or not (P1). Conversely, a transient increase of IgM could be associated with an increase in EDSS (P4, P5), or not (P1, P8). In P8, it can be noted that the important drop of EDSS (from 4 to 0) was associated with an increase in IgM levels. Overall, these data suggest that there is no evident correlation between the evolution over time of ETX-specific IgM response and the variations of EDSS.

## 3. Discussion

The hypothesis that *C. perfringens* ETX might be an environmental trigger for MS was raised after *C. perfringens* type B was isolated for the first time from a patient at clinical presentation of MS [16]. This finding, together with the known CNS-tropism of *C. perfringens* ETX and binding to oligodendrocytes/myelin, prompted the initiation of serological surveys to assess the immunoreactivity against ETX in MS patients. The survey performed by Rumah et al. in a US population [16] reported immunoreactivity to ETX in about 10% of people with MS and 1% of healthy controls. Another investigation in a UK population of clinically definite multiple sclerosis, performed by Wagley et al. [17], detected seroreactivity to ETX in 24% of the patients and 10% of matched healthy controls. In that study, seroreactivity was also tested against linear overlapping peptides spanning the amino acid sequence of ETX, where 33% of patients’ sera reacted to at least one peptide, as compared to 16% in the control group [17]. These two serological surveys used conventional Western blot techniques with methodological limitations for the detection of low-abundance proteins and limited specificity. Against this background, we used a sensitive quantitative ELISA assay to determine the isotype pattern and concentrations of anti-ETX antibodies in sera from untreated RRMS patients and matched controls.

First, using Western blotting, our data confirmed the reactivity to ETX of sera from RRMS patients. However, the overall incidence was higher (65%) than the incidence reported in previous studies [16,17] and the incidence was just as high in the control group. The discrepant results of ETX antibodies in patient’s sera by Western blotting are likely related to the different methods used in the distinct studies, including different amounts of ETX antigen and different serum dilutions. In addition, ETX-specific antibodies had no neutralizing activity, in agreement with previous findings [17]. Thus, the detection of antibodies that react to ETX in healthy controls suggests that this reactivity does not characterize an abnormal response linked to MS. The failure of the antibodies to neutralize ETX argues against a toxin-specific antibody response. Indeed, it was reported that *C. perfringens* ETX induces high levels of neutralizing antibodies, either during natural human infection with *C. perfringens* [32] or after immunization of rabbits, sheep, or cattle with *C. perfringens* epsilon-toxin vaccine candidate [33]. Moreover, it is noteworthy that low concentrations of native or recombinant ETX antigen are sufficient to induce high levels of neutralizing antibodies [34,35].

By assessing the isotypic pattern and serum concentration of ETX-reacting antibodies, our study brings new findings that reveal a predominant IgM response over IgG and IgA antibody responses both in MS patients and controls and, moreover, significantly higher levels of IgM reacting to ETX in MS patients compared to controls. As a whole, these findings were unexpected and not consistent with a presumed ETX-specific response. Assuming that ETX production is episodic, due to brief cycles of *C. perfringens* growth followed by long periods of quiescence, as suggested by Ma et al. [18], a dominant IgG antibody response should be detected in MS patients. On the other hand, Huss et al. investigated, by direct detection, the occurrence of ETX and anti-ETX antibodies in MS patients, but neither ETX nor antibodies against it were detected in serum samples, arguing against the hypothesis of a causal relationship between *C. perfringens* ETX and MS [36].

Unspecific immunoreactivity against various antigens is known to occur in MS patients and does not necessarily indicate a primary immune response against a disease related agent [37]. Identified target antigens include neurotropic viruses, such as measles or varicella zoster virus [38], microorganisms such as Chlamydia pneumoniae [39], as well as various self-antigens partly directed against intracellular autoantigens released during tissue destruction [40]. Lipids that are considerably present in the CNS have also been identified as target antigens. In particular, intrathecal IgM recognizing anti-myelin lipids have been detected in MS patients, able to activate complement dependent demyelination and representing a predictor of aggressive evolution in MS [41]. In this line, serum IgM to phosphatidylcholine has recently been identified as a new diagnosis biomarker in RRMS patients and a predictive marker of response to treatment [42,43]. Against this background, we believe that the predominant and long-lasting IgM response that we report in RRMS patients, together with the low affinity and lack of neutralizing activity of the antibodies, is more in favor of a cross-reactivity with an auto-antigen than an ETX-specific immunoreactivity. Autoantibodies are also present in healthy individuals [44,45]. They accumulate throughout life, increase with age [46], and some of them, particularly IgM, are characterized by poly-affinity for various antigens. Natural poly-specific self-reactive IgM antibodies may exert a spectrum of effects from injurious to protective depending upon cellular and molecular context, a role in the regulation of some anti-inflammatory responses [47,48].

Finally, our findings of high prevalence of anti-ETX antibodies in MS patients as well as in HCs contrast with the very low carriage of ETX-producing *C. perfringens* in humans. ETX-producing *C. perfringens* have been detected by a sensitive PCR method in a greater abundance in fecal samples of MS patients compared to healthy people, but at infinitesimal levels. It was speculated that this pathogen could be quiescent for long periods and undergo brief growth phases with ETX production [18]. Despite this, ETX-producing *C. perfringens* was rarely isolated from humans [16,32,49], although it is frequently found in animal species such as sheep [19]. It is noteworthy that MS is more common in urban environments than in rural areas, where the source of human contamination with *C. perfringens* is more accessible [50].

ETX recognizes myelin and lymphocyte protein (MAL) as specific receptor [29,30], which is distributed on endothelial cells in the central nervous system (CNS), oligodendrocytes, peripheral nerve Schwann cells, and mature human T lymphocytes but not on murine T cells [50]. A hallmark of ETX biological activity is ETX targeting oligodendrocytes leading to demyelination, as shown in rat and mice tissue and cell models [26,28,51]. Experimental autoimmune encephalomyelitis (EAE) has been developed in mice and rats and ETX-induced EAE represents a closely related model of MS [18]. ETX can cross the blood brain barrier likely via caveolae-dependent transcytosis through brain endothelial cells and to gain access to brain tissues as investigated in experimental animals upon intravenous ETX administration [25,52]. However, ETX affinity to cells expressing human MAL is about ten times less than cells overexpressing rat of sheep MAL [29,33], suggesting that humans can only develop a mild or chronic form but not an acute ETX disease [21]. However, despite a high ETX affinity for rat MAL, no naturally acquired ETX-induced disease was reported in rats or mice. An initial and crucial step in ETX-producing *C. perfringens* disease is the production of ETX in the intestinal content and passage through the intestinal barrier. Typically, overgrowth of ETX-producing *C. perfringens* in sheep intestinal content subsequent to sudden feeding of large amount of starch-rich food is accompanied by high production of ETX, which passes through the intestinal barrier, disseminates via blood circulation, and enters the CNS leading to characteristic central nervous signs of excitation [19,27]. In contrast, goats, which share an identical MAL protein sequence with sheep MAL (Supplementary Figure S1), mainly develop enterocolitis. It is speculated that ETX is absorbed more readily from sheep intestine than of goats [23,53]. ETX increases intestinal permeability in rats and mice [54], but ETX absorption, through the intestinal barrier according to diverse animal species and humans, remains to be defined. ETX has not yet been detected in fecal and other biological samples of MS patients, suggesting that the ETX antibodies in MS and HCs might result from a cross-reacting antigen with ETX. Moreover, no neutralizing anti-ETX antibodies have been reported in MS patients ([16,17,36] and our study), though ETX-based vaccines induce strong toxin neutralizing response and ETX targets human lymphocytes [33,55].

Gut dysbiosis in MS patients has been identified in numerous studies. Common findings of fecal microbial DNA/RNA investigations in MS consisted of higher ratios of Firmicutes/Bacteroidetes with higher abundance of Streptococcus genus versus lower prevalence of Prevotella genus. However, no increase in Clostridia in the microbiota of MS patients has been shown in these studies [56,57,58,59]. The role of gut dysbiosis in MS is not fully understood. However, bacterial metabolites such as short-chain fatty acids and bile acids influence the host immune responses [56]. Gut dysbiosis has been associated not only with MS but with various autoimmune diseases [60]. In this overall context, we believe that the high prevalence of seroreactivity to ETX recognized in MS patients is more compatible with a cross immunological reactivity with an antigen, possibly an autoantigen mimicking ETX, than a toxin-specific response. MS is a complex and heterogeneous disease, traditionally categorized by distinct clinical features, but a new consideration of the course of MS was proposed as a spectrum defined by the relative contributions of overlapping pathological and reparative/compensatory processes [4]. According to Tanja Kuhlmann et al. [4], moving from clinically-based to biologically-based definition of MS progression would contribute to a better understanding of key mechanisms underlying progression and help the implementation of measures to quantify progressive pathology.

## 4. Conclusions

MS is a multifactorial disease characterized by chronic-immune neurological disorder with progressive demyelination and neuronal cell loss in the central nervous system. *C. perfringens* ETX is a potent toxin that is able to cross the blood brain barrier, and which targets oligodendrocytes and certain neuronal cells by interacting with the MLA receptor. ETX induces demyelination and is responsible for a severe neurological disease in animals, mainly in lambs. ETX has been proposed to be a causative agent of MS in humans. Our investigation on immunological response to ETX by Western blotting and quantitative ELISA shows a high prevalence of anti-ETX antibodies, predominantly IgM over IgG and IgA, in the sera of one hundred MS patients as well as in ninety healthy controls. The wide distribution of anti-ETX antibodies in MS patients as well as in healthy controls supports a cross reactivity of ETX with another antigen, likely an autoantigen, rather than a specific response to ETX as causative agent of MS.

## 5. Material and Methods

### 5.1. Patients and Serum Samples

Human biological samples and associated data were obtained from NeuroBioTec (CRB-HCL Hospices Civils de Lyon Biobank BB-0033-00046) and are part of a collection declared at the French Department of Research (DC 2008-72). Informed consent was obtained from all participants. This prospective observational study included patients (n = 100) with relapsing remitting multiple sclerosis (RRMS). Longitudinal serum samples were available at baseline and 4 times over a 3-to-5-year follow-up for 10 patients. None of these patients were treated at the time of study. Healthy controls (n = 90) were selected based on the inclusion criteria of no diagnosis of MS or other neurological disease. They were collected and provided by the Institut Pasteur’s Biological Resources Centre (CRBIP). Demographic data and clinical characteristics of patients with MS and healthy controls are described in Table 1.

### 5.2. Toxin

*C. perfringens* epsilon prototoxin was produced from *C. perfringens* strain 15246 and purified as previously described [61]. Epsilon prototoxin was activated with TPCK-treated bovine trypsin (Sigma-Aldrich, Sigma Aldrich Chimie S.a.r.l, 38297 Saint-Quentin-Fallavier Cedex, France) for 1 h at room temperature.

### 5.3. Western Blotting

One μg of ETX was loaded in each well of an SDS-polyacrylamide (10%) gel and then transferred onto nitrocellulose membranes, which were blocked in phosphate-buffered saline containing 0.1% Tween20 (PBST) and 5% dried milk. The membranes were incubated with the sera diluted 1000-fold in PBST overnight at room temperature and then washed with PBST. Detection was performed with goat anti human IgG-peroxidase (Sigma A0170, Sigma Aldrich Chimie S.a.r.l, 38297 Saint-Quentin-Fallavier Cedex, France) 1:3000 in PBST for 1 h at room temperature and enhanced chemiluminescence (Thermo Fisher Scientific, SCI Duguay-Trouin, 44800 Saint-Herblain, France).

### 5.4. Quantitative ELISA for Epsilon Toxin-Serum and Peptide Antibody Responses

Epsilon toxin or TGVSLTTSYSFANTN peptide (Pepscan, 8243 RC Lelystad, The Netherlands) were used as antigens. Sera were tested by ELISA using a quantitative assay, as previously described [62]. Briefly, 96-well plates (MaxiSorp, Nunc) were coated overnight at 37 °C with 100 µL of epsilon toxin (4 µg/mL) or the peptide (30 µg/mL) diluted in the coating buffer (carbonate/bicarbonate 0.1% *v*/*v* sodium deoxycholate, pH 9.6). Plates were then washed with the washing buffer (PBS containing 0.1 to 0.5% (*v*/*v*) Tween^®^20 (Sigma) to remove unbound antigen. Unsaturated sites were blocked with 200 µL of the saturating buffer (PBS 3% BSA) 1 h at 37 °C. Sera to be tested were diluted with the Multiprobe II^®^ robot in the dilution buffer (PBS 1% BSA 0.1 to 0.5% (*v*/*v*) Tween^®^20), and then 100 µL of diluted serum were added in each well and incubated for 1 h at 37 °C. The plates were washed with the washing buffer. Goat anti-human IgG, IgA, or IgM alkaline phosphatase-conjugated antibodies were added and antigen-specific antibodies detected with para-nitrophenyl phosphate (pNPP) substrate. The reaction was then stopped by 100 µL of sodium hydroxide and the optical density was read at 405 nm. The calibration curves were set up using purified polyclonal human IgG, IgA, and IgM (Sigma). The wells were coated with serial dilutions of the corresponding isotypes, ranging from 1.7 to 46 ng/mL for IgM, 0.1 to 18 ng/mL for IgG, and 2 to 70 ng/mL for IgA. The assay was then performed as described above, and the concentrations of serum antibodies were calculated according to the calibration curves. Serum Ig concentrations are expressed in μg/mL.

### 5.5. Neutralization Assay

Madin–Darby canine kidney (MDCK) cells were grown in Dulbecco’s modified Eagle medium (DMEM) supplemented with 10% fetal bovine serum. Confluent cells grown on 96 well plates were incubated with serial dilutions of ETX and propidium iodide (PI) (5 g/mL). At 3 and 18 h, the plates were read with a spectrofluorometer (Fluoroskan II) (excitation 380 nm and emission 620 nm). The results were expressed as the percentage of fluorescence obtained compared to non-intoxicated cells incubated with 0.5% Triton X100 at 37 °C. The last ETX dilution yielding 50% cytotoxicity was considered to contain 1 cytotoxic unit (CU). Sera of MS patients were diluted 1:10 in DMEM containing 4 CU of ETX and PI in a final volume of 100 L and incubated 1 h at 37 °C. Then the mixtures were added to MDCK cells and the lectures were performed at 3 and 18 h.

### 5.6. Statistical Analysis

Statistical analysis was performed using GraphPad Prism version 9 (GraphPad software, San Diego, CA, USA). Nonparametric measures of associations were used, including the Mann–Whitney *U*-test, the Wilcoxon signed rank test, linear regression, and Spearman rank correlation. Values of *p* < 0.05 were considered statistically significant.

### 5.7. Ethics Statement

The study was conducted in accordance with French law relative to clinical non-interventional research. According to the French law on Bioethics (29 July 1994; 6 August 2004; and 7 July 2011, Public Health Code), the patients’ written informed consent was collected. Moreover, data confidentiality was ensured in accordance with the recommendations of the French commission for data protection (Commission Nationale Informatique et Liberté, CNIL decision DR-2014-558).

## Figures and Tables

**Table 1:** Demographic data and clinical characteristics of the study population.

Study Subjects	n	Female n (%)	MS Subtype	Age at First Sampling (Y) Median (Range)	Disease Duration (Y) (Median, Range)
All MS	100	77 (77.0)	RRMS	36 (18–58)	8.3 (0–32.5)
P1	1	F	RRMS	47	16.7
P2	1	F	RRMS	41	11.3
P3	1	F	RRMS	33	8.3
P4	1	F	RRMS	35	14.3
P5	1	F	RRMS	25	4.3
P6	1	F	RRMS	30	7.5
P7	1	F	RRMS	21	5
P8	1	F	RRMS	21	0.5
P9	1	F	RRMS	32	3.3
P10	1	F	RRMS	41	5.4
Healthy controls	90	47 (52.0)	NA	44 (20–78)	NA

**Table 2:** Immunoreactivity of the sera with ETX by Western blotting.

Immunoreactivity	Sera from MS Patients n (%)	Sera from Healthy Controls n (%)
strong	10 (14.3%)	10 (11.1%)
medium	14 (15.7%)	36 (40.0%)
weak	34 (38.2%)	24 (26.6%)
negative	31 (34.8%)	20 (22.2%)
Total positive/total number	58/89 (65.1%)	70/90 (77.7%)

## Data Availability

The original contributions presented in this study are included in the article/Supplementary Materials. Further inquiries can be directed to the corresponding author.

## Supplementary Materials

The following supporting information can be downloaded at: https://www.mdpi.com/article/10.3390/toxins17010027/s1, Figure S1: Alignment of MAL protein sequences from sheep (XP_004006224) and goat (XP_017910309). Extracellular loops 1 (ECL1) and 2 (ECL2) which are likely binding receptor sites for ETX according to Rumah, KR, Y Ma, JR Linden, ML Oo, J Anrather, N Schaeren-Wiemers, MA Alonso, VA Fischetti, MS McClain and T Vartanian. The Myelin and Lymphocyte Protein MAL Is Required for Binding and Activity of *Clostridium perfringens* epsilon-Toxin. PLoS Pathog 2015;11:e1004896. [29].
