# Supplementary material for: Does Clostridium Perfringens Epsilon Toxin Mimic an Auto-Antigen Involved in Multiple Sclerosis?
Source: Toxins (Basel). 2025 Jan 7;17(1):27. doi: 10.3390/toxins17010027 (PMC11768822; doi:10.3390/toxins17010027)
Supplement: Supplementary file 1 [file toxins-17-00027-s001.zip › toxins-3345699-supplementary.pdf]

Supplementary Materials: Does Clostridium Perfringens Epsilon Toxin Mimic an Auto-Antigen Involved in Multiple Sclerosis?

Marie-Lise Gougeon, Valérie Seffer, Cezarela Hoxha, Elisabeth Maillart and Michel R. Popoff

```
                                                    ECL1
sheep   1 mapsaasgvsslpsgfavfttfpdllfifefvfgglvwilvssshvpipliqgwvmfasv  60
          ||||||||||||||||||||||||||||||||||||||||||||||||||||||||||||
goat    1 mapsaasgvsslpsgfavfttfpdllfifefvfgglvwilvssshvpipliqgwvmfasv  60


sheep  61 fcfvattvlaflyvigahgnrtswitldaayhcvaalfyfgasvlealvtielqdgffyk  120
          ||||||||||||||||||||||||||||||||||||||||||||||||||| ||||||||
goat   61 fcfvattvlaflyvigahgnrtswitldaayhcvaalfyfgasvlealvtiqlqdgffyk  120


sheep 121 yyhenisavvfsyvatllyvvhavfslirwkss  153   ECL2
          |||||||||||||||||||||||||||||||||
goat  121 yyhenisavvfsyvatllyvvhavfslirwkss  153
```

Figure S1. Alignment of MAL protein sequences from sheep (XP_004006224) and goat (XP_017910309). Extracellular loops 1 (ECL1) and 2 (ECL2) which are likely binding receptor sites for ETX according to Rumah, KR, Y Ma, JR Linden, ML Oo, J Anrather, N Schaeren-Wiemers, MA Alonso, VA Fischetti, MS McClain and T Vartanian. The Myelin and Lymphocyte Protein MAL Is Required for Binding and Activity of Clostridium perfringens epsilon-Toxin. PLoS Pathog 2015;11:e1004896. [29]
